# Supplementary material for: Causal Relationship between Aging and Anorexia Nervosa: A White-Matter-Microstructure-Mediated Mendelian Randomization Analysis
Source: Biomedicines. 2024 Aug 16;12(8):1874. doi: 10.3390/biomedicines12081874 (PMC11351342; doi:10.3390/biomedicines12081874)
Supplement: Supplementary file 1 [file biomedicines-12-01874-s001.zip › Supplementary Figure/Figure S4/Singe SNP plot of MR analysis for TL to ubm-b-1734.pdf]

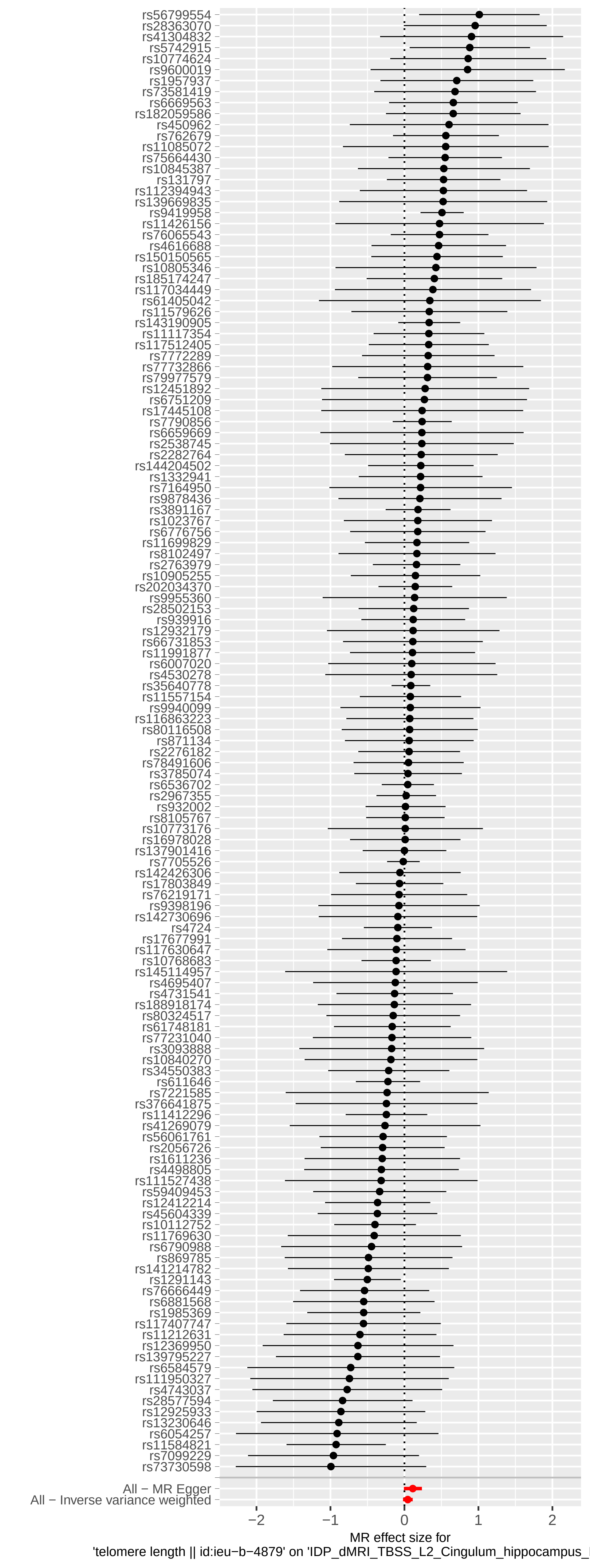
rs56799554
rs28363070
rs41304832
rs5742915
rs10774624
rs9600019
rs1957937
rs73581419
rs6669563
rs182059586
rs450962
rs762679
rs11085072
rs75664430
rs10845387
rs131797
rs112394943
rs139669835
rs9419958
rs11426156
rs76065543
rs4616688
rs150150565
rs10805346
rs185174247
rs117034449
rs61405042
rs11579626
rs143190905
rs11117354
rs117512405
rs7772289
rs77732866
rs79977579
rs12451892
rs6751209
rs17445108
rs7790856
rs6659669
rs2538745
rs2282764
rs144204502
rs1332941
rs7164950
rs9878436
rs3891167
rs1023767
rs6776756
rs11699829
rs8102497
rs2763979
rs10905255
rs202034370
rs9955360
rs28502153
rs939916
rs12932179
rs66731853
rs11991877
rs6007020
rs4530278
rs35640778
rs11557154
rs9940099
rs116863223
rs80116508
rs871134
rs2276182
rs78491606
rs3785074
rs6536702
rs2967355
rs932002
rs8105767
rs10773176
rs16978028
rs137901416
rs7705526
rs142426306
rs17803849
rs76219171
rs9398196
rs142730696
rs4724
rs17677991
rs117630647
rs10768683
rs145114957
rs4695407
rs4731541
rs188918174
rs80324517
rs61748181
rs77231040
rs3093888
rs10840270
rs34550383
rs611646
rs7221585
rs376641875
rs11412296
rs41269079
rs56061761
rs2056726
rs1611236
rs4498805
rs111527438
rs59409453
rs12412214
rs45604339
rs10112752
rs11769630
rs6790988
rs869785
rs141214782
rs1291143
rs76666449
rs6881568
rs1985369
rs117407747
rs11212631
rs12369950
rs139795227
rs6584579
rs111950327
rs4743037
rs28577594
rs12925933
rs13230646
rs6054257
rs11584821
rs7099229
rs73730598
All − MR Egger
All − Inverse variance weighted
−2
−1
0
1
2
MR effect size for
'telomere length || id:ieu−b−4879' on 'IDP_dMRI_TBSS_L2_Cingulum_hippocampus_l
